# The Post-Traumatic Growth Journey of Women Who Have Survived Intimate Partner Violence: A Synthesized Theory Emphasizing Obstacles and Facilitating Factors

**DOI:** 10.3390/ijerph19148653

**Published:** 2022-07-16

**Authors:** Hulda S. Bryngeirsdottir, Denise Saint Arnault, Sigridur Halldorsdottir

**Affiliations:** School of Health Sciences, University of Akureyri, Solborg v/Nordurslod, 600 Akureyri, Iceland; starnaul@med.umich.edu (D.S.A.); sigridur@unak.is (S.H.)

**Keywords:** post-traumatic growth (PTG), intimate partner violence (IPV), gender-based violence (GBV), mental health, trauma recovery, rehabilitation, women’s health, public health, theory development, theory synthesis

## Abstract

Suffering intimate partner violence (IPV) is a devastating personal experience and post-traumatic growth (PTG) is a positive, psychological change in a person, following trauma such as IPV. There is a gap in the literature when it comes to theories on PTG after surviving IPV. The aim of this theory development was to synthesize an approach to understanding the PTG journey of female IPV survivors. According to our theory, their PTG journey includes eight main components: 1. The women’s early experience of trauma, 2. The consequences of that trauma, 3. Their experiences of IPV, 4. The consequences of IPV, 5. The facilitating factors to PTG, 6. The hindering factors to PTG, 7. Their experience of PTG, and 8. The lingering effects of IPV. According to our findings, PTG is a real possibility for female IPV survivors, and it is likely to improve their mental health, well-being, and quality of life, as well as that of their children, loved ones, and communities, thereby decreasing the damaging effects of IPV. The theory can be useful for professionals when guiding female survivors of IPV to promote their recovery and healing. Due to the lack of research in this field, additional research is needed to further develop this theory.

## 1. Introduction

Gender-based violence (GBV) is a serious, societal problem [1,2,3,4,5], affecting approximately one in three women all around the world [1,2,3]. The forms of GBV vary and the causes are multidimensional, including social, cultural, economic, and political [5,6]. Intimate partner violence (IPV) is the most common type of violence against women [1,2,3,5,7] and includes physical and psychological aggression, controlling behaviour and/or sexual coercion, the perpetrator most often being a current or former intimate partner [2,7]. Global research has revealed that women are more likely to be assaulted, injured, raped, or killed by their male spouse or male ex-spouse than by anyone else [8,9,10]. Even though the typologies of IPV vary [11,12,13], the consequences of suffering IPV are often serious, affecting the woman’s physical and psychological health in a destructive way [2,5,14,15,16,17,18,19] and even resulting in her being murdered by the perpetrator [15,20]. Suffering IPV also affects the victim’s social wellbeing in a negative way [4,5,9,21], as well as that of her children [4,5,7,9,21,22] and loved ones [5,9,21,23]. Women who have suffered IPV endure a higher illness burden, their comorbidity is high, they are more likely to be diagnosed with mental illness and are in increased danger of substance use disorders than women who have not experienced IPV or abuse [7,15,24].

Violence against women is now widely considered a serious public health problem [7,21,25] which concerns all sectors of society and violates human rights [2,7,21,26,27]. According to WHO’s study on women’s health and domestic violence against women, conducted in fifteen settings in ten countries, sociodemographic factors, such as age, marital status, and educational status, did not explain the differences found between the settings of the research [21]. GBV, including IPV, has severe economic costs not only for the victims of violence, but also for their community. These costs are due to expenditures on service provision because of violence, lost income for the women suffering GBV/IPV and their families, decreased productivity, as well as the destructive impacts on future human capital formation, affecting the economic growth in a negative way [27]. Economic abuse is a unique form of abuse, well-known within intimate partner relationships, which negatively affects the victim’s life in an extensive way, i.e., her mental health and psychological well-being, family formations, parenting practices, children’s behavior, and youth outcomes [28].

A considerable proportion of people experience psychological trauma sometime in their lives. The key aspects of psychological trauma are life threat, uncontrollability, and unpredictability [29,30]. Even though a traumatic experience can lead to various psychological problems [31,32,33], most trauma survivors show enormous adaptability when coping with their experience [34,35]. The development of post-traumatic outcomes depends on the physical and emotional proximity to the traumatic event [33,36,37,38]. Suffering IPV is a complex traumatic experience [39,40], in which the perpetrator has forced the victim into survival mode by taking over the control of her life [39]. Therefore, making the decision of staying in or leaving a violent relationship is also complex [41,42]. Leaving such a relationship means profound changes in the life of the female survivor of IPV, since the woman moves from survival mode to starting a new life where she is in control [39]. In a qualitative online survey, 665 female survivors of IPV described their experiences and definitions of their long-term recovery following IPV. When defining their recovery, the women focused on their lived experience of the phenomenon instead of psychological and academic concepts commonly used by researchers. The five themes they used to describe their definition of recovery were safety and survival, having their freedom, moving on, having a better life, and issues with children and parenting. Many of them also described relapses, digressions, and highs and lows as a part of their recovery. The themes described were woven together in their description of their journey to recovery. According to these findings, recovery following IPV can take a long time and is both individual and multidimensional in nature, requiring a great deal of support [43].

Post-traumatic growth (PTG) has been described as a positive, psychological change in a person, following traumatic events and severe difficulties, where the person focuses on the possible positive outcomes of the trauma instead of focusing on the negative consequences [44]. PTG consists of five main components, i.e., the person experiences positive spiritual change, sees new possibilities in life, appreciates life more, experiences increased personal strength, and has better relations to other people [44,45]. Research has shown that many people who have had symptoms of PTSD following trauma, have described these extensive positive changes in their lives [46,47,48,49,50,51,52,53,54]. When estimating PTG, all these components are considered [50,51,52,53,54,55]. We identified a gap in the literature since most PTG work has been undertaken on a variety of trauma and little is known about PTG after IPV. The general nature of PTG theory can be criticized when being used for various groups of trauma survivors, such as for female survivors of IPV and, therefore, trauma-specific PTG theories are needed.

Recognizing IPV as a major social problem that negatively affects public health has progressively changed attitudes toward IPV against women [25,56]. This has resulted in an increased interest in the research area of IPV [25,56], leading to an international, steady increase in the number of publications on the subject over the last 20 years [25]. Research on PTG has been conducted in various fields of the literature of trauma, such as transportation accidents or other accidental injuries [57,58,59], natural disasters [60,61,62,63,64,65], interpersonal experiences [66,67,68,69,70,71,72,73,74,75,76,77,78], medical problems [79,80,81,82,83,84,85,86], and other life experiences [87,88,89,90]. Even so, when it comes to research on PTG following IPV among female survivors, there seems to be a severe lack of literature [91]. Since PTG has been shown to improve quality of life in multiple ways, the possibility of PTG among female survivors of IPV is important.

### Purpose of the Theory Development and the Main Question

This theory development is part of a larger research project aimed at exploring the post-traumatic growth of female IPV survivors. The first study in the research project was a phenomenological study about the experience of PTG among people who had suffered various traumas [92], and the following two phenomenological studies examined the facilitators [93] and obstacles to female survivors’ PTG journey following IPV [94]. The main aim of the present theory development is identifying and describing the main components of the PTG journey of female IPV survivors. Our full team consisted of two professors (one Icelandic and one American) as well as one Icelandic doctoral student. The Icelandic team conducted the phenomenological and theoretical analysis. We developed the theory through theory synthesis using the key concepts and key statements reported in our already published papers, our rich databases on the subject, as well as published material pertaining to PTG in IPV survivors. When organizing existing knowledge into a framework about a certain phenomenon, combining it with databases of the phenomena of interest to develop a theory on the subject, theory synthesis is an appropriate and well-known methodological strategy [95]. Therefore, when developing this theory, the theory synthesis method was used. The purpose of the theory development was to identify, describe, and explain the main components on the PTG journey of Icelandic female IPV survivors. The main question of the theory development was: What are the main components of the PTG journey of female IPV survivors?

## 2. Materials and Methods

### 2.1. Design of the Theory Development

After pulling together available knowledge on the components of the PTG journey of female IPV survivors in our already published studies, our databases of the subject, as well as published material pertaining to PTG in IPV survivors, key concepts, and key statements were organized into a synthesized theory, mainly by using abstract thought processes, e.g., critical, conceptual, and creative thinking, as well as inductive and deductive reasoning. Since only a part of our research data has already been published, we have access to a large amount of data that we have collected on the phenomenon, which gave us the opportunity to present an even deeper understanding of the PTG journey of female IPV survivors. However, we only use findings where more than half of the twenty-two participants in the databases of the studies [92,93,94] reported a key component. When undertaking various studies on the same phenomena from different angles, researchers can gain more insight on the research data by using the research results from the new studies, which is the aim of theory synthesis [95].

### 2.2. The Method of Theory Synthesis

The method of theory synthesis involves three steps, where, in step one, the key concepts and key statements of the synthesized theory are specified. In step two, the literature is reviewed to identify factors that relate to the key concepts and key statements. In step three, the key concepts and key statements are then organized into an integrated description of the phenomena [95]. The three main steps of the theory synthesis, and the way it was used in this theory development, are described further in Table 1. 

### 2.3. Steps in the Theory Synthesis

Step 1. The bases of the theory are the lived experiences of female survivors on their journey to PTG following IPV. When describing their PTG journey, they reported their traumatic experiences earlier in life, as well as the facilitators and obstacles affecting their journey to PTG. The women’s experience of PTG and the lingering effects of IPV on their growth were also reported. An overview of the studies and research data used in the first step of the theory synthesis can be found in Table 2 and Table 3.

Step 2. After working through the evidence base constructed in the first step of the theory synthesis, we analyzed the results of the studies, along with the academic writing used to form the literature background in our own studies in the field of post-traumatic growth following intimate partner violence. To deepen the understanding of the phenomena, we repeatedly examined our research data on the subject. This process was undertaken so that we could come to a joint conclusion about the female survivors’ journey to PTG following IPV, including the influencing factors on that journey and the lingering effects of IPV on their PTG. By conducting this analysis, we found confirmation of our findings in step 1. We found that PTG is possible for IPV survivors, despite their lived experience of IPV. Former experience of traumatic events earlier in life should, however, be considered when processing the experience of IPV, aiming for PTG, as well as the facilitators and obstacles met by the survivors of IPV that affect their journey to PTG. It is likely that when a female survivor of IPV reaches PTG, she also experiences some lingering, negative effects of her experience of IPV, even if enjoying PTG. 

Step 3. In this last step, we present the results using methods that are the most appropriate for the subject. We chose to present the theory by using text, figures, and tables.

## 3. Results

The primary question our theory answers is “What are the main components of the PTG journey of female IPV survivors”? When presenting the results, we begin with identifying and defining the main concepts of the theory, i.e., trauma, intimate partner violence (IPV), facilitators of PTG, obstacles to PTG, post-traumatic growth (PTG), and lingering effects of IPV (see Table 4). Secondly, we describe and explain the essence of the theory through the eight major components of the PTG journey of female IPV survivors, identified by the authors i.e., 1. Life before IPV, 2. Wounded or adapted? 3. The experience of IPV, 4. IPV’s consequences, 5. Main facilitating factors on the PTG journey, 6. Main obstacles on the PTG journey, 7. The experience of post-traumatic growth and, finally, 8. The lingering effects of IPV. We used text, tables, and figures to present the theory. These are presented in Section 3.1.1, Section 3.1.2, Section 3.1.3, Section 3.1.4, Section 3.1.5, Section 3.1.6, Section 3.1.7 and Section 3.1.8.

### 3.1. Description of the Theory

**Figure 1:**
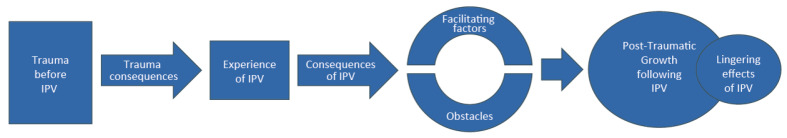
The eight main components of the post-traumatic growth journey by female survivors of intimate partner violence. *Note*. The figure, developed by the authors for the present theory, introduces the eight main components of the PTG journey as experienced by female IPV survivors. 1. Trauma before IPV. 2. Trauma consequences. 3. Experience of IPV. 4. Consequences of IPV. 5. Obstacles on the PTG journey. 6. Facilitating factors on the PTG journey. 7. Post-traumatic growth following IPV. 8. Lingering effects of IPV. Part of the facilitating factors are likely to be part of the women’s PTG, while part of the obstacles is likely to remain as part of the negative effects of IPV lingering into her PTG. Though pictured as a one-way, linear process, PTG is a nonlinear, fluid state, where regression, e.g., due to triggers, should be considered. Even so, a female survivor of IPV is likely to be aware of the possibility of regression in her PTG, as well as knowing the best ways for her to respond to such regression. According to this, reaching PTG is not a permanent condition, but requires the woman enjoying it to constantly maintain and nourish her growth.

This theory aims to answer the main question by explaining main components of the PTG journey among female survivors of IPV. The theory includes the effects of the trauma and violence they endured early in life, and how that experience served as a certain preparation for their later life experiences. The women’s experience of IPV, as well as the consequences they suffered because of it, are considered when theorizing about the PTG journey, as well as the facilitators and obstacles. Some women enjoy more facilitators on their journey, while others meet more obstacles, affecting their possibilities of reaching PTG. The theory includes the survivors’ experience of PTG while considering the lingering effects of IPV on their lives after reaching PTG. Though pictured as a one-way process, PTG is a nonlinear, fluid state and regression, e.g., due to triggers, should be considered. Even so, a woman enjoying PTG seems to know how to react to regression in her PTG. She seems to be aware of the possibility of regression, knowing what to be aware of in that matter. She also seems to know the best ways to react to her regression, being aware of that she will overcome this bad feeling and regain her wellbeing, enjoying her PTG. A woman enjoying PTG is also aware of the importance of maintaining it. An overview of the theory is shown in Figure 1.

#### 3.1.1. Component 1: Trauma before IPV

We theorize, based on the aforementioned findings, that a substantial majority of women who suffer IPV have already suffered traumatic events, as children and/or young adults. When experiencing violence and traumas as a child, an adolescent, or a young adult, the female victim has neither the power nor the control in her life, as she would have as an adult, making her more vulnerable and fragile. She, however, reacts in the best way she can, to survive. Suffering traumas early in life is likely to produce serious long-term effects on the victim’s selfhood, resulting in fragile boundaries and vulnerability. Some women, however, seem to adapt to their traumatic situation and their experience of suffering trauma early in life, feeling as if they wear a certain shield for their protection. The feeling of being protected by a shield seems to result in their avoidance in confronting demanding situations, instead ignoring them, accepting them as their unchangeable reality. In some cases, however, the ‘snowball’ effects of previous traumas that have not been processed; this can gradually make survivors of traumas more vulnerable as the size of the ‘ball’ grows, resulting in a traumatic break-down. We theorize from our findings that if traumatic experiences are not processed in a constructive way, they are likely to undermine the future reaction of the woman when facing further trauma and violence, such as IPV, leaving her more vulnerable than before. 

#### 3.1.2. Component 2: Influences of Former Traumas on the Experience of IPV

We theorize, based on the aforementioned findings, that the results of traumatic experiences early in life appear as the victims move on with their lives, either through carrying their vulnerable selfhood and deconstructed boundaries, or through having adapted, feeling stronger, better protected, and even better prepared for life. We theorize that in both cases, the women’s personal boundaries have been moved, twisted, damaged, or broken, leaving their personhood fragile. Even if prior experience of traumatic events can motivate and increase the inner strength of the survivors, we propose that whether the woman feels vulnerable or adapted, she is more likely to experience difficulties in confronting traumas and violence later in life, thus being in great danger of being violated in many ways as an adult, including IPV. 

An overview of the traumas the women had suffered prior to their experience of IPV and their long-term negative consequences are shown in Table 5.

#### 3.1.3. Component 3: Experience of IPV

We theorize, based on the aforementioned findings, that the woman in a violent relationship often feels trapped. To survive, she gradually moves her personal boundaries, resulting in the man gaining full power over her life. We theorize that the woman often denies that she is in a violent relationship, hiding what is really going on, thus does not seek help and, therefore, no one is able to help her. Female survivors’ experiences of IPV according to the theory are described in Table 6.

#### 3.1.4. Component 4: Consequences of IPV

We theorize, based on the aforementioned findings, that IPV negatively affects the woman’s well-being, health, and life. The trauma process is unique and individual, not only influencing the survivor, but also her children, her loved ones, and her community. Leaving a violent relationship is a complicated, exhausting, and time-consuming process, the completion of it taking place gradually over time. After leaving the perpetrator, the woman often confronts various negative feelings, as well as health problems and poor social status due to her experience of IPV. All this can result in her loss of working capacity, which again leads to loss of routine, loss of social interaction with others, and loss of income. Thus, the post-IPV trauma effects involve various negative influences on the woman’s physical and mental health, as well as her social wellbeing, leaving her even more vulnerable. An overview of the consequences of IPV according to the theory is shown in Table 7. 

#### 3.1.5. Component 5: Obstacles on the PTG Journey

We theorize, based on the aforementioned findings, that the experience of IPV generally has severe, destructive effects on the survivor’s life, creating various obstacles on their PTG journey [94]. We propose that women often suffer negative and diverse feelings towards themselves, experience fear or have been conditioned through their experience of being in a violent relationship, which often prevents them from reporting the violence and seeking appropriate help and support, serving as an obstacle in their PTG. This said, help-seeking is a complex and multifaceted phenomenon, and we do not want to oversimplify it. Women who have survived IPV are often easily triggered; those who have children often suffer on behalf of their children. The perpetrator is likely to keep on abusing and harassing them and their children in various ways, they often feel lonely, tend to overreact, and frequently experience difficulties in emotional connection to other people. All these factors are likely to influence their health and well-being in negative ways, serving as obstacles on their PTG journey. Being in a relationship where the perpetrator is in charge and constantly needs to be pleased is a lot of work. This often results in the woman not taking care of her health; she cannot rest and constantly feels anxious and alert, resulting in a constant decline in her wellbeing. After being in such long-term, stressful situations of a violent relationship, many female survivors of IPV suffer severe long-term or chronic consequences. After being in the stressful situations of a violent relationship for a long-term period, many female survivors of IPV suffer severe health problems, often leading to a loss of working capacity, thus undermining their social welfare and creating obstacles on their PTG journey. The perpetrator often continues harassing the woman after their relationship has ended. Having the perpetrator constantly reminding her of his presence can be very confusing for the woman, reducing her chances of being able to let go of him and depriving her of the peace to recover, serving as an obstacle to her PTG.

According to our theory, many female survivors of IPV feel powerless when it comes to the laws, regulations, and social system; they may experience that the perpetrator holds all the power of the social system in his hands, as well as of the laws and regulations. If not divorced, the woman does not receive the benefits nor the financial support that she is entitled to by law as a single mother, even though she is providing for the children. The perpetrator has many ways in which to use the law, regulations, and social system to delay the separation or divorce, as well as the division of assets and custody of children. Since the woman is forced by law to let the perpetrator meet with the children, even against the children’s will, the perpetrator often uses the children to control the woman. Waiting to have the power over their lives, having the perpetrator continuously harassing them in various ways, as well as experiencing the feeling of powerlessness towards laws, regulations, and the social system, affect the woman’s mental and physical health in a negative way, often leaving them in an even more vulnerable position towards the perpetrator than before, thus serving as an obstacle on the women’s PTG journey. An overview of the obstacles on the journey to PTG following IPV is shown in Table 8. 

#### 3.1.6. Component 6: Facilitators on the PTG Journey 

We theorize, based on the aforementioned findings, that there are various facilitators for the women’s PTG journey following IPV [93]. Survivors who demonstrate positive personal competence, along with personal skills they have advanced through their experiences in life, seem to be better qualified to process their experience of IPV and move along their way to PTG. When a woman makes an independent decision of changing her circumstances for the better and seeks help, it serves as a facilitator of her PTG. When doing so, she is likely to reconsider her perspective to herself, work on her courage, confront and process her experiences of IPV, and set goals for a better future, which are likely to facilitate her PTG. To live in secure circumstances, enjoying social welfare, being able to meet her and her children’s basic needs, is an essential facilitating factor in the woman’s PTG, since it gives her the opportunity to concentrate on other things than mere survival. Possessing earlier experience of trauma can serve as a helpful preparation for some survivors of IPV. When followed by effective trauma processing, the former experience of trauma can serve as a promoter, increasing the woman’s inner personal coping skills as well as her strength, which is likely to facilitate her PTG. 

We theorize, based on the aforementioned findings, that it is most effective when the woman herself decides where to seek help, taking the time she needs to work on her task, being self-compassionate while doing so, and reviewing her attitudes towards herself and the ways she treats herself. One of the facilitators on the PTG journey seems to be for the woman to consider and work on her perspectives regarding her loved ones and other people, setting boundaries as well as encouraging good relations where possible. By considering her perspectives to her loved ones and other people, and vice versa, the woman can analyze the patterns of communication with others, deciding if her relations with others are healthy and helpful for her or if she needs to set some boundaries to be in control of her life. The woman setting boundaries to the perpetrator is also an important task for her, to seize and hold on to the control of her own life, serving as a facilitator of her PTG. We theorize that the woman experiencing personal support from her surroundings, as well as from other resources, when building a better life following IPV is a valuable facilitating component on her PTG journey. According to our theory, the most valuable support provided is the support that meets the personal needs of the woman; getting the help she needs, when she needs it, as well as communicating with kind, respectful, and supportive people when dealing with the consequences of IPV and working her way towards PTG. This personal support can be in various forms: informal, formal, or organized resources, or all these types. The absence of the health care system in most of the participating women’s accounts reflects their experience that they did not expect the health care system to intervene to facilitate their PTG.

Overview of the facilitators on the journey to PTG following IPV is shown in Table 9. 

#### 3.1.7. Component 7: Post-Traumatic Growth Following IPV

**Figure 2:**
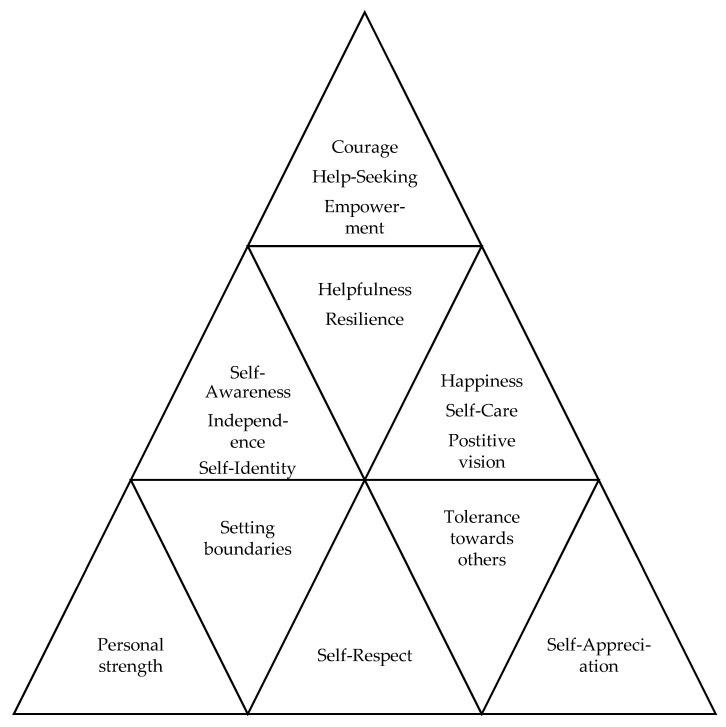
Experience of post-traumatic growth by female survivors of intimate partner violence. *Note*. The figure, developed by the authors as part of the present theory, shows the main concepts the women used to describe their experience of PTG following IPV. The most common concepts are at the bottom of the triangle, serving as a foundation for the descriptive concepts above. The second most common concepts used to illustrate PTG following IPV are in the next row above, building an additional support for the next row above, etc. In accordance with this figure, most of these descriptive concepts are intrapersonal, illustrating that women who enjoy PTG following IPV see themselves in a positive way and have respectful attitudes towards themselves. The figure also contains some interpersonal concepts, referring to the women being respectful of themselves and helpful to others as part of their PTG.

We theorize based on the before-mentioned findings that suffering and surviving terrible violence like IPV can result in various positive outcomes for female survivors, the women being able to view themselves, their lives, and their prospects in a more positive and constructive way than before. Their experience of PTG following IPV is described by various explanatory concepts, most of them being intrapersonal, i.e., existing or occurring within themselves or in their minds, while only a few of the concepts used in describing PTG are interpersonal, i.e., occurring between persons, or are both intrapersonal and interpersonal. When perceiving PTG, the women sense their increased inner strength and self-respect, where they appreciate themselves more and set boundaries to self and others to guard their self-identity. The women seem to know themselves better and are more tolerant towards other people, feeling free, complete, and happy at the same time. They appear to take good care of themselves, looking forward to their future and want to do good by using their experience to help other survivors of IPV. The women are likely to feel resilient and in charge of their lives, not hesitating in seeking appropriate help when they need it and seem to be fully aware of what they need and what they want for themselves. An overview of the experience of PTG by female survivors of IPV is shown in Figure 2. 

#### 3.1.8. Component 8: Lingering Effects of IPV in PTG

We theorize, based on the aforementioned findings, that PTG is not a permanent condition but needs to be continuously nourished and maintained by the woman enjoying it. Despite all the positive effects of PTG on the woman’s life, the complicated effects of IPV often appear to be long-lasting, serving as lingering negative effects on the PTG of female IPV survivors. While enjoying PTG, many survivors seem to continue experiencing various triggers, bringing back the memories of the violent situation that they were stuck in, causing them “heavy days” and discomfort. Ongoing communication with the perpetrator as well as experiencing their children’s suffering because of the violent relationship are also likely to have negative lingering effects on their PTG, as well as their various health problems following the violent relationship, which often have an extensive negative effect on their quality of life. After suffering IPV, women often lose confidence and trust in other people, causing them difficulties in establishing and maintaining relationships with others, also negatively affecting romantic relationships, even after having reached PTG.

**Figure 3:**
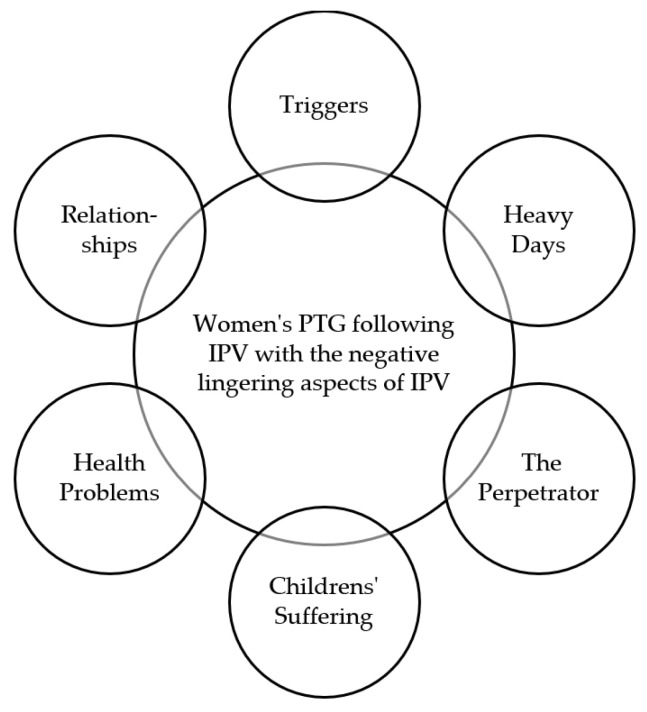
Component 8: An overview of the lingering effects of IPV in female survivors’ PTG. *Note*: The figure, developed by the authors for the present theory, shows the negative lingering effects of IPV affecting female survivors’ PTG. The figure illustrates women’s PTG following IPV in the middle of the figure, where the effects of IPV are lingering from the sides into the area of PTG. Despite the lingering effects of IPV, the survivors’ PTG seems to be strong, since the women enjoying PTG are not letting the effects of IPV overtake their PTG. They are aware of the lingering effects of IPV, know how to handle the situation, and are still enjoying PTG.

We theorize, based on the aforementioned findings, that female survivors of IPV who have reached PTG are aware of the lingering effects of IPV on their PTG. Having reached PTG, they are likely to be aware that these negative effects of IPV can appear, but they seem to know how to react to them, and are aware that these lingering effects of IPV on their PTG are not permanent. The women seem to be aware of their capability to process the lingering effects of IPV without letting go of their PTG. An overview of the lingering negative effects of IPV in female survivors’ PTG is shown in Figure 3.

## 4. Discussion

Our theory introduced in this article is a valuable contribution to the field of research on PTG and IPV. The purpose of our theory development was to answer the main question, what are the main components of the PTG journey of female IPV survivors? by identifying, describing, and explaining the main components of the PTG journey of female IPV survivors. We theorize that the PTG journey of female survivors of IPV consists of eight main components, as explained in the findings.

When reflecting on people’s reaction to suffering personal traumas, research results have revealed various risk factors, as well as protective factors. These influencing factors have been divided into three categories: pre-traumatic, i.e., former experience of trauma; peritraumatic, i.e., severity of the trauma; and post-traumatic, i.e., the person’s reaction to the trauma as well as the reaction of his or her surroundings. Social support is helpful in all these stages [96,97]. Our theory is in accordance with this division of influencing factors regarding the reaction to trauma supports, where the woman’s life before IPV influences both the probability of her entering a violent relationship as well as her experiences of the violence and how she reacts to the violent situation. According to our theory, the most important facilitating factors on the woman’s PTG journey are her intrapersonal attributes, even though social support is always helpful. 

According to our theory, the woman’s life as a child and/or a young adult is likely to affect the way she is prepared for life. Experiencing trauma and violence early in life, either leaves her vulnerable, where she must move or deconstruct her boundaries to survive; or she adapts to her traumatic situation, resulting in her avoiding confronting difficult situations, but accepting them instead, feeling strong. Either way, to survive, the woman is likely to have changed and deconstructed her basic human rights, leaving her more vulnerable than before. If she is fortunate, she comes to know and interact with genuine and good people in life, but we note that women with prior traumatic experience are in danger of becoming the perfect prey for perpetrators of violence and abuse. Accumulated loads of prior traumas can result in a “building block effect”, thus increasing the probability of negative psychological outcomes in victims of traumas [98,99]. Accordingly, the characters and future defenses to traumatic events as adults, as well as their psychological outcomes, can be linked to the women’s childhood experiences of trauma. Notably, the results of a recent study of adverse childhood experiences (ACE) and mental health among women experiencing IPV, suggest that IPV survivors are more likely to have multiple and severe ACEs [100].

Research results suggest that, when suffering more than one kind of trauma, it is more likely to affect the victim’s mental and physical wellbeing in a negative way, than when suffering one kind of trauma [101]. However, the severity of the consequences depends on how personal the traumatic event is and how intimate the perpetrator is with the victim [33,36,37,38,101]. Accordingly, being violated against and traumatized by one’s intimate partner has a great negative effect on the person’s welfare, and repeated violent behaviour is likely to gradually decrease the victim’s well-being, health, and quality of life. Each female survivor of IPV suffers and processes her trauma in an individual and unique way [43], which not only influences the surviving woman, but also her children, her loved ones, and her community. 

According to our theory, female survivors of IPV meet various obstacles as well as facilitating factors on their PTG journey. Those with more facilitators and less obstacles are more likely to move forward on the PTG journey. Loss of working capacity is one of the reasons behind the financial problems that are very common among female survivors of IPV, leading to various other problems, such as lack of housing and other necessities, undermining their welfare [28]. The experience of financial problems is not only due to the loss of the women’s working capacity, but to the financial abuse during their violent relationship, which often continues even if the relationship has ended, sometimes resulting in long-term poverty and even the woman’s bankruptcy [28].

Our theory supports the findings of a recent qualitative study; 18 Australian women in the age range of 50–69, who had left an abusive relationship, were interviewed about their experiences of IPV at different stages of their lives. They reported that being in the violent relationship directly affected their physical, psychological, and financial wellbeing in an extensive way. During the period of separation, many women experienced continuing abuse as well as stress due to housing, legal matters, and financial difficulties. After the separation they felt lonely and traumatized, their economy was weak, and they had problems due to damaged relationships with other people [102]. In a recent mini-review of gender-based violence during the COVID-19 pandemic, the research results revealed that the legislatures and services available for victims of IPV are often insufficient, thus worsening their situations [103].

The absence of the health care system in the findings may suggest a need for improvement in that system.

The theory is in line with the results of a recent systematic review of the facilitators of the recovery from GBV, that showed that to recover it is important for the woman to reconnect with themselves, their surroundings, and the world in general, by having support from both formal and informal networks, as well as from other people. According to this systematic review, it is important not to blame the woman, to emphasize the possibilities for her to change her situation, and to address and work on her reflection to affect intimate relationships [104].

PTG has been defined as positive, psychological change in a person, focusing on the possible positive outcomes following traumatic experience [44,73]. According to our theory, suffering and surviving IPV can result in various positive outcomes for the woman, such as reaching PTG. We suggest that a great part of PTG in female IPV survivors emerges in their personal, inner growth and the reconstruction of themselves. The foundation of their PTG is intrapersonal, i.e., possessing positive feelings and respectful attitudes towards themselves and taking the actions needed to preserve that attitude and to be in control of their lives. When enjoying PTG, the women seem to know themselves better and experience various positive feelings towards themselves, feeling resilient and in charge of their lives. They seem to know what they want, know what they need and seek appropriate support when needed. Although their experience of PTG is likely to increase tolerance towards other people, they do not hesitate to set boundaries at the same time, to protect their self-identity and the control of their own lives. 

According to our theory, proposed here, even though reaching PTG following the experience of IPV is a great achievement for female survivors, it is neither a simple nor a permanent condition. Life goes on, with its ups and downs, and the survivors’ life is not always perfect. Survivors of IPV have experienced serious traumas and their being has been systematically undermined in their violent relationships [1] and the effects of IPV linger into their PTG. The ‘triggers’ seem to be all around in their environment and many of the women endure ‘heavy days’ in between, where they are feeling down, going through complicated and negative feelings and discomfort in relation to their experience of IPV. 

We theorize that, the frequently continuing harassment of the perpetrator is making their life hard, the women often suffer on behalf of their children’s endurance of the former and often ongoing violence, and the women’s health problems are likely to extensively affect their lives in a negative way [102]. In addition to all these factors, female survivors of IPV often have trouble trusting other people, which diminishes the possibilities of them having healthy romantic relationships. According to our theory, these negative effects of IPV are likely to linger into their PTG; however, having reached PTG, survivors of IPV seem to be able to recognize these lingering effects of IPV and find the best ways to process them without losing their PTG.

### 4.1. Limitation of the Theory Development

In step 1 of the theory development we theorized from our three published papers [92,93,94]. Moreover, we used dense raw data consisting of approximately 300,000 words. However, a large part of that data is unpublished, which is a limitation.

### 4.2. Future Research and Theory Development Directions

Due to the lack of research in the field of PTG among female IPV survivors, more research is needed to further develop the theory. More research would not only advance the theory but also its specific components. Those undertaking studies requiring a theory as a research framework could use this theory as a framework. Finally, new theories are needed on the PTG journey of IPV survivors, e.g., from the perspective of men and intersex people.

## 5. Conclusions

When it comes to theory development in the field of PTG among female IPV survivors, there is a gap in the literature. Our research contributes to that field in an important way by undertaking theory development on the eight main components of the PTG journey of female IPV survivors, emphasizing the main obstacles and facilitators on that journey. According to our theory, PTG is a real possibility for female survivors of IPV, which is likely to result in their increased wellbeing and quality of life, as well as the wellbeing of their children and loved ones, and the community as whole, minimizing the destructive effects of IPV. Due to the high prevalence and serious consequences of IPV, it is important to provide some potential of a better life for the survivors. The absence of the health care system in the findings may suggest a need for improvement in that system. Aiming for PTG after suffering IPV is important. The information provided in the theory could be useful for the public and professionals when guiding female IPV survivors on the path to better lives, promoting their recovery and healing, aiming for PTG.

## Figures and Tables

**Table 1:** Theory synthesis: an overview of the method and how it was used in this theory development.

Step	Description	Overview of What We Did
Step 1	The key concepts and key statements from the studies and the databases, used to develop the theory, are specified and explained.	We used our own studies and extensive databases (see Table 2 and Table 3) and analyses of them in the theory synthesis. These contain information about how female survivors of IPV who had reached PTG described their journey to PTG, how they perceived their PTG, and how the lingering effects of their former traumatic experience influenced their PTG.
Step 2	The key concepts and key statements used to develop the theory are compared to the literature, to identify and define their relation to other factors.	The key concepts and key statements identified in step one were used when comparing the main concepts used in the theory to the literature of PTG among female survivors of IPV. Most of the articles from the literature were partially related to the women’s journey to PTG, their experience of PTG, and the lingering effects of their prior traumatic experience in life on their experience of PTG.
Step 3	The key concepts and key statements of the theory and their relations are presented in text, figure(s), or table(s).	After comparing the detailed descriptions of female survivors’ journey to PTG following IPV, their experience of the facilitators and the obstacles on the journey as well as of PTG and the lingering effects of their prior traumatic experience on their PTG. We present the results in text, figures, and tables.

**Table 2:** The published studies used to develop the theory in step I.

Authors and Date	Title	Published
Bryngeirsdottir and Halldors-dottir, 2021 [92]	The Challenging Journey from Trauma to Post-Traumatic Growth: Experiences of Facilitating and Hindering Factors	Scandinavian Journal of Caring Sciences 00, 1–17
Bryngeirsdottir and Halldors-dottir, 2022 [93]	“I’m a Winner, Not a Victim”: The Facilitating Factors of Post-Traumatic Growth among Women Who Have Suffered Intimate Partner Violence	International Journal of Environmental Research and Public Health 19, 1342. Special Issue: Environment and Behavior
Bryngeirsdottir and Halldors-dottir, 2022 [94]	Fourteen Main Obstacles on the Journey to Post-Traumatic Growth as Experienced by Female Survivors of Intimate Partner Violence	International Journal of Environmental Research and Public Health 19, 5377. Special Issue: Violence against Women as an Interdisciplinary Challenge in Public Health

**Table 3:** Summary of research data collected by the first author, used to develop the theory in step I.

Research Data	Number of Interviews	Main Criteria for Participation	Word Count
Qualitative interviews	13	Icelandic men and women who self-reported PTG following traumas	90.172 (M = 6.936)
Qualitative interviews	22	Icelandic female IPV survivors who self-reported PTG following traumas caused by IPV	199.386 (M = 9.063)
SUMMARY	35 interviews		289.558 (M = 8.273)

**Table 4:** The main concepts of the theory—defined by the authors for the theory.

Concepts	Definitions
Trauma	An unexpected and threatening event experienced by an individual that he or she cannot stop, control, or influence in any way. Trauma negatively affects the basic experience of living in a safe and predictable world and can even negatively affect the individual’s worldview.
Intimate Partner Violence (IPV)	Controlling, dominating and/or violent behaviors in an intimate relationship that causes the victim physical, psychological, sexual, financial, or social harm.
Facilitators of PTG	Personal, social and/or systematic constructive components that are likely to be beneficial to the progress of PTG among female survivors of IPV. For example, these may be internal factors of the woman, i.e., personal abilities, mindset, social wellbeing, former experience of trauma; the attitude and reaction of the woman, the perpetrator, children, loved ones, and other people; and environmental factors, i.e., personal social support, systematic social support, and organized supporting resources.
Obstacles to PTG	Personal, social, and/or systematic destructive components, which are likely to cause a delay in, or prevent, the progress of PTG among female survivors of IPV. These are, for example, feelings of shame; suicidal thoughts; fragile self-identity; insecurity; feeling alone and isolated; triggers; mixed negative feelings; emotional connection to others; physical and psychological health; personal circumstances and social surroundings; the perpetrator; the children; and law and the institutional social system.
Post-Traumatic Growth (PTG)	Following the experience of trauma and through the individual’s internal need for change, the woman has managed to process the suffering caused by the trauma. The personal changes experienced include confronting one’s own feelings more freely, consciously nourishing inner strength, having deeper relations to others, experiencing personal growth, living a more wholesome life, and having deeper self-knowledge as well as a stronger self-image. Furthermore, the individual enjoys increased social activity, positivity, and patience and has feelings of freedom, power, and energy, without any regrets. Moreover, the individual feels like a winner in life, is less stressed, more appreciative of one’s own self, others, and life in general, seeing new possibilities in life, having found a new vision as well as deeper inner peace and wisdom. Even though the negative influences of trauma can be present, the positive factors of post-traumatic growth are dominant. Post-traumatic growth can be likened to a personal resurrection in life following psychological trauma.
Lingering Effects of IPV	The negative, long-term effects of traumatic experience are intertwined with one’s PTG. The person becomes aware of these effects, learns to accept them and how to endure them, responding to them in the best and most suitable way, knowing that the effects will pass and/or everything will be all right.

**Table 5:** *Component 2*: Overview of the former traumas the participants in the studies had experienced and their long-term negative consequences.

Former Traumas as a Child or Young Adult	Negative Results of Former Traumas	Influence of Former Traumas on Reacting to Traumatic Situations	Influences of Former Traumas on IPV
As a child Neglect, poverty, sexual abuse, bullying, witnessing IPV at home, alcohol abuse by parents, illness, or death of a relative, dependent atmosphere at home, parents’ divorce, apathetic and absent parents, demanding parents, stigmatization by community (i.e., because of adverse conditions at home), taking on too much responsibility for their age, difficulties at school. As a young adult Violent relationship, rape, bullying, assault, oppression, threats, property damage, breach of confidentiality, infidelity, divorce, custody dispute, neglect of children, post-partum depression, sickness of loved ones, death of loved ones, financial concerns, accidents, loss of health, codependence, drug abuse by herself or former spouse, alcohol abuse by the survivor or former spouse, bankruptcy.	Fragile self-image, lower feelings of self-worth, shift in personal boundaries, depressed defensive responses, diminished trust in other people, dependence, excessive feeling of responsibility, shame, anxiety, perfectionism, rebelliousness, forbidden to complain, having to succeed no matter what, insecurity, feeling of rejection, grief, suicidal attempts, muscle tension, fear, stress, feeling of guilt, sleep problems, reticence, nervous breakdown.	Destructive reaction to traumas, trouble in processing trauma in a constructive way. Feeling vulnerable or adapted to traumatic situations avoiding confronting the real situations. Snowball-effects of past and current traumas sometimes ending in traumatic breakdown.	Increased danger of being abused, reducing possibilities of leaving violent and life-threatening situations.

**Table 6:** Component 3: Overview of experiencing IPV.

	The Female Survivors’ Lived Experience of IPV: A Summary
Female IPV Survivors	We theorize, based on the aforementioned findings, that being a female victim of IPV can be compared to being held as a hostage in a violent relationship against your will. The woman feels captured and dependent on the perpetrator, where most things are conditional, him deciding what is “right” and what is “wrong”, and her “bad behavior” having serious, unpredictable consequences. The woman often feels like she is being silenced, since her opinion does not matter, her words do not have meaning, her needs are ignored, and her will and reactions to the situation seem not to be relevant. Due to the perpetrator’s gaslighting, as well as his unpredictable mood and behaviour, the woman often becomes exhausted when trying to please the perpetrator. She seems to continuously move and reset her personal boundaries, losing a small piece of her self-identity every time she does so. In the end, her boundaries are likely to be completely shattered; the woman experiences complete vulnerability and hopelessness, and gives up. Often, she cannot choose whom she meets, she cannot confide in anyone, and there is no one left to back her up or defend her. The perpetrator often has full access to her whenever he wants, threatening her and abusing her in the ways he pleases. Even though the woman is likely to be terrified, she cannot expect anyone to come and rescue her since the violent situation is frequently concealed; the woman may feel like she has been sworn to secrecy and that no one can know of the violence. The woman’s physical and psychological health is often systematically threatened as well as her wellbeing. In the end, she is likely to suffer serious health problems if the situation is either long-term or even permanent.

**Table 7:** Component 4: Overview of the intrapersonal and interpersonal consequences of IPV, according to the women participating in the studies.

Intrapersonal Consequences of IPV	Interpersonal Consequences of IPV
Experiences feelings of fear, grief, anger, shame, helplessness, and betrayal. Feeling of not being herself anymore, having been conquered, defeated, and overpowered. Experiences fear of acknowledging the violence. Easily triggered, feels tired, stressed, suicidal, feels like she has lost so much, and feels uncertain about the future. Suffers insomnia due to anxiety and fear, never knows what will happen next, feels insecure, lacks appetite, suffers pain due to physical injuries. Feels like someone is constantly watching her, feels ashamed of letting the relationship go on for so long. Experiences difficulties in performing usual activities of daily life.	Experiences social isolation, has stopped seeing friends, has stopped seeing family, and has stopped communicating with other people. Does not know how to behave, fakes her feelings, fakes her wellbeing, and pretends to be happy. Feels emotionally absent to other people, experiences lack of interest in sex, as well as lack of interest in romantic relationships

**Table 8:** Component 5: Overview of the main obstacles on the women’s PTG journey following IPV according to the theory.

Main Obstacles	Examples
Negative feelings towards own self	Feels ashamed, blames herself, feeling of being less worthy, experiences self-stigmatization, suicidal thoughts, injured self-identity, disrupted body image, insecurity, anger, and loneliness.
Triggers	Incidents related to the experience of IPV that negatively affect the woman’s feelings and wellbeing, e.g., sees a car that resembles the perpetrator’s car, reads a column in the paper that diminishes victims of IPV.
Conflicting states of mind	Experiences relief vs. regret, strength vs. vulnerability, joy vs. misery, and comfort vs. displeasure.
Negative feelings on behalf of their children	Feels sad because of what the children have endured due to the violent relationship. Feels angry because of continuing destructive behavior of the perpetrator towards the children.
Problems in connecting to other people	Experiences lack of trust, avoidance of emotional connections, fear of romantic relations and loss of own social standards. Often overreacts to other people’s behaviours, actions, words, mimics, tone of voice and body posture.
Health issues	Feels tired, in pain, has trouble sleeping, feels tense, depressed, anxious, endures physical diseases, physical and/or mental breakdown, and burnout.
Challenging personal circumstances	Experiences lack of housing, financial problems, loss of working capability, and social isolation.
Self-destructive behaviour	Talks to herself in a hostile and hurtful way. Blames herself for her situation.
The perpetrator	Continues harassing, stalking, showing threatening, frightening, violent behaviour, financial abuse, and escalating psychological violence.
Mixed feelings towards the perpetrator	Has nightmares, experiences flashbacks and fear, finds it hard to let go. Can be obsessed with the man.
Negative feelings towards laws, regulations, and the social support system	Feels powerless within ‘the system’, the divorce/separation takes a long time, the division of assets is unfair, the man stays in control, the woman is forced to settle with the perpetrator about their assets and children, she is forced to send the children to the perpetrator against their will, experiences fear of child protection services taking her children away, the perpetrator uses the children to blackmail the woman, while still married to the perpetrator or cohabitated with him, by law, the woman does not receive the support and benefits that she is entitled to as a single mother.

**Table 9:** Component 6: Overview of the main facilitators on the women’s PTG journey following IPV, according to the theory.

Main Facilitators	Examples
Personal competence and skills	Positive attitude, personal strength, and resilience.
State of mind	Confronting the experience of violence. Rejecting current situation. Deciding to seek help. Setting goals for a better life and PTG. Taking control of own life. Deciding not to be a victim.
Social welfare	Safe living conditions. Safe place to live. Financial security. Professional support.
Previous experience of trauma	Earlier experiences of processing trauma, resulting in increased inner strength
Self-perspective	Chooses where to seek help. Has self-compassion. Gives herself the time needed. Treats herself right
Perspective to loved ones and other people	Considers behaviour towards others and the behaviour from others. Encourages good relations. Sets boundaries.
Perspective to the perpetrator	Sets boundaries. Prevents him from being in control.
Various personal support	Informal support. Systematic support. Organized resources.

## Data Availability

The data presented in this theory development are with the corresponding author. Because of anonymity as well as ethical and personal reasons, the raw data are not available, but the published data are all available in open-access format.
